# Metabolic Dysfunction-Associated Steatohepatitis and Progression to Hepatocellular Carcinoma: A Literature Review

**DOI:** 10.3390/cancers16061214

**Published:** 2024-03-20

**Authors:** Haider Ghazanfar, Nismat Javed, Abeer Qasim, George Sarin Zacharia, Ali Ghazanfar, Abhilasha Jyala, Elona Shehi, Harish Patel

**Affiliations:** 1 Division of Gastroenterology, Department of Internal Medicine, BronxCare Health System, Bronx, NY 10457, USAajyala@bronxcare.org (A.J.); eshehi@bronxcare.org (E.S.); 2 Department of Internal Medicine, BronxCare Health System, Bronx, NY 10457, USAgzachari@bronxcare.org (G.S.Z.); 3 Department of Internal Medicine, Fauji Foundation Hospital, Rawalpindi 45000, Pakistan

**Keywords:** MASH, HCC, pathogenesis, progression, risk factors

## Abstract

**Simple Summary:**

The global prevalence of metabolic-associated fatty liver disease (MAFLD) is rising due to urbanization, obesity, poor diet, sedentary lifestyles, and genetic factors. We conducted a narrative review on MAFLD and hepatocellular carcinoma (HCC) risk factors, pathogenesis, and biomarkers using databases. Steatosis, an early stage of MASH progression, is linked to metabolic syndrome factors like obesity and type 2 diabetes. Mechanisms such as increased lipolysis and hepatic lipogenesis contribute to liver lipid accumulation, exacerbated by insulin resistance. Natural compounds show promise in regulating lipid metabolism and inflammation. Liver fibrosis predicts MASH and HCC development, emphasizing its importance in treatment strategies. Risk factors for MASH-associated HCC include advanced liver fibrosis, older age, male gender, metabolic syndrome, genetics, and dietary habits, highlighting the need for effective surveillance and diagnostics. Further studies are needed to understand the biochemical impact of these risk factors for targeted therapies to prevent HCC or reduce HCC risk.

**Abstract:**

The prevalence of metabolic-associated fatty liver disease (MAFLD) is increasing globally due to factors such as urbanization, obesity, poor nutrition, sedentary lifestyles, healthcare accessibility, diagnostic advancements, and genetic influences. Research on MAFLD and HCC risk factors, pathogenesis, and biomarkers has been conducted through a narrative review of relevant studies, with a focus on PubMed and Web of Science databases and exclusion criteria based on article availability and language. Steatosis marks the early stage of MASH advancement, commonly associated with factors of metabolic syndrome such as obesity and type 2 diabetes. Various mechanisms, including heightened lipolysis, hepatic lipogenesis, and consumption of high-calorie diets, contribute to the accumulation of lipids in the liver. Insulin resistance is pivotal in the development of steatosis, as it leads to the release of free fatty acids from adipose tissue. Natural compounds hold promise in regulating lipid metabolism and inflammation to combat these conditions. Liver fibrosis serves as a significant predictor of MASH progression and HCC development, underscoring the need to target fibrosis in treatment approaches. Risk factors for MASH-associated HCC encompass advanced liver fibrosis, older age, male gender, metabolic syndrome, genetic predispositions, and dietary habits, emphasizing the requirement for efficient surveillance and diagnostic measures. Considering these factors, it is important for further studies to determine the biochemical impact of these risk factors in order to establish targeted therapies that can prevent the development of HCC or reduce progression of MASH, indirectly decreasing the risk of HCC.

## 1. Introduction

The incidence and prevalence of metabolic-associated fatty liver disease (MAFLD) have steadily increased across the globe. Urbanization, obesity, nutrition, physical inactivity, access to healthcare, and advancements in diagnostic methods and genetic factors might have contributed to the disease burden. The estimated global prevalence of MAFLD among adults is approximately 30%, more so in males than in females [1,2,3,4]. Meta-analyses from Asia and Europe report an MAFLD prevalence of roughly 30% [1,2,3,4]. Most studies conclude that continental South America has the highest prevalence of MAFLD, estimated to be as high as 59%, while Africa has the lowest prevalence, reported at around 13.5% [3,5,6,7]. In North America, the National Health and Nutrition Examination Surveys from 2017–2018 reported an overall prevalence of 56.7%, while Raizi et al. reported 47.8% [3,8]. MAFLD is most frequent amongst Hispanics, followed by non-Hispanic whites, and least in non-Hispanic blacks [3,8]. The estimated global incidence of MAFLD per 1000 person-years ranges from 46.13 to 52.34 but varies across regions [1,2,3,7]. Parallel to MAFLD, metabolic dysfunction-associated steatohepatitis (MASH) is also on the rise, with an estimated overall prevalence of about 5.27%, while it is 5% in North America [9]. An estimated 80–100 million Americans may have MAFLD, and MASH is the second most common indication to live transplant in the United States [10]. Hepatocellular carcinoma (HCC) is considered the sixth most common cancer, and owing to its dismal overall prognosis, the third most common cause of cancer-related mortality worldwide [11]. A recent study from the United States reported an HCC prevalence of 4.6 and 374.4 per 10,000 persons amongst non-cirrhotic and cirrhotic MAFLD patients, respectively [12]. Younossi et al. estimate annual HCC incidence rates of 0.44 and 5.29 per 1000 person-years, with MAFLD and MASH, respectively [11,13]. According to the American Gastroenterology Association, the incidence of HCC in MAFLD-related cirrhosis is >1.5% per year, justifying HCC surveillance in this subset of patients [10]. Mathematical models predict MAFLD/MASH to be a significant health problem, with a 122% increase in MAFLD-related HCC by 2030 [14]. The objective of the review is to elaborate the possible mechanisms responsible for progression to HCC from MASH and the associated risk factors.

## 2. Materials and Methods

A narrative review was conducted using PubMed and Web of Science using the following keywords: “hepatocellular carcinoma”, “metabolic dysfunction-associated steatohepatitis”, “risk factors”, “non-alcoholic”, “pathogenesis”, and “biomarkers”. The studies were screened using the abstract to retrieve full articles. Abstracts without a full article or not in English were excluded. English translations were obtained for articles included that were in different languages.

## 3. Review and Discussion

**Figure 1:**
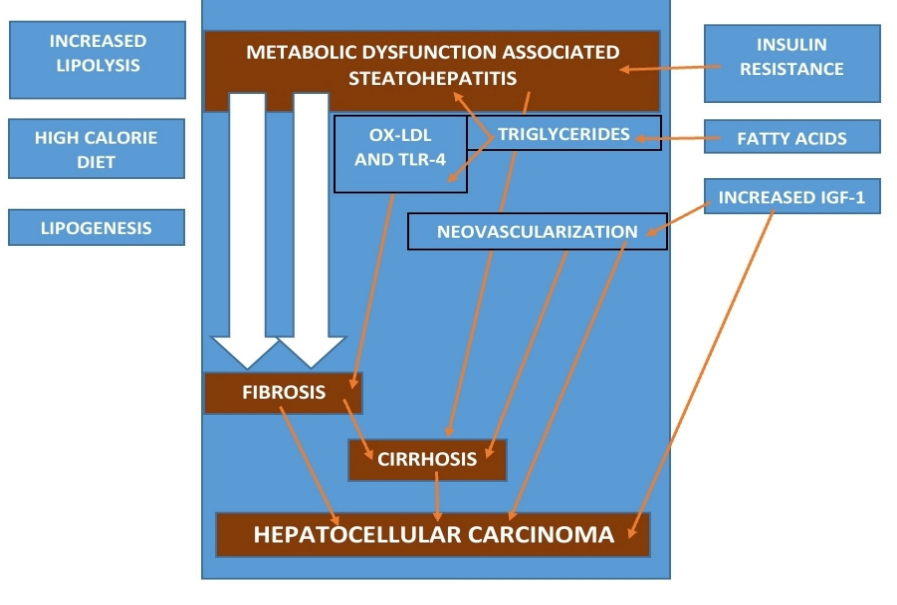
Hypothetical model of a hepatocyte showing possible pathogenic mechanisms.

A summary of the pathogenesis and associated risk factors is illustrated in Figure 1.

### 3.1. Pathogenesis

Steatosis, characterized by fat accumulation in liver cells, occurs when the intake or production of fat surpasses its breakdown or elimination. This marks the initial stage in the progression of MASH, often associated with metabolic syndrome features like obesity, type 2 diabetes, dyslipidemia, and hypertension [13]. Three main mechanisms contribute to excessive lipid buildup in the liver: increased lipolysis in visceral adipose tissue (AT), activation of hepatic de novo lipogenesis (DNL), and consumption of high-calorie/fat diets [15]. Insulin resistance (IR) plays a pivotal role in hepatic steatosis development by rendering AT resistant to insulin’s antilipolytic effects, leading to increased release of free fatty acids (FFAs) that accumulate as TGs in the liver [16].

Additionally, dietary factors exacerbate IR and contribute to MAFLD progression. Sugary foods, particularly those high in fructose, also promote steatosis by directly influencing lipid metabolism [17]. Furthermore, IR is linked to hepatocellular carcinoma (HCC) development, as evidenced in animal models and human studies. Insulin and insulin-like growth factor (IGF)-1 stimulate cell proliferation and inhibit apoptosis, fostering carcinogenesis [18]. High glucose levels and sustained hyperglycemia further contribute to HCC development through inflammatory signaling cascades and the generation of reactive oxygen species (ROS) [18]. Moreover, IR can accelerate hepatocarcinogenesis by promoting hepatic neovascularization [19].

While triglycerides are a predominant component of liver lipids in both MASH and simple steatosis, they pose minimal lipotoxicity risk, as they serve as safe storage lipids. However, other lipid molecules such as cholesterol, free fatty acids (FFAs) and their derivatives, diacylglycerols, and ceramides are implicated in lipotoxicity [20]. Lipotoxicity exerts cellular damage through three main mechanisms. Firstly, harmful lipids disrupt the function of intracellular organelles like the endoplasmic reticulum and mitochondria; secondly, they directly alter intracellular signaling pathways, such as by increasing ceramide levels, thereby affecting metabolic and inflammatory pathways; thirdly, interactions between lipids in the cell surface or cytoplasm and cellular kinases indirectly modify signaling, leading to inflammation and other biological effects [21].

Lipotoxicity-induced hepatocyte death correlates with the severity of MAFLD. Increased lipid peroxidation can activate macrophages by generating ligands for scavenger receptors like oxidized low-density lipoprotein (ox-LDL) [22]. Additionally, saturated fatty acids stimulating TLR4 is another mechanism of macrophage activation, further exacerbating the hepatic inflammatory response [23]. Crucially, macrophage-mediated stimulation of surviving hepatocytes through pathways like NF-kB and other cell proliferation pathways is a significant aspect of hepatocarcinogenesis [23].

Disruption of protein folding processes induces ER stress, where unfolded or misfolded proteins accumulate, triggering the unfolded protein response (UPR) to restore ER homeostasis [24]. However, prolonged ER stress activates apoptotic pathways, leading to cell death. The UPR involves three signaling pathways mediated by PERK, IRE1, and ATF6 [24]. These pathways regulate lipid metabolism, with XBP1 and eIF2α influencing lipid regulation. ER stress can induce hepatic steatosis by reducing VLDL synthesis and promoting lipid droplet formation [24]. Furthermore, ER stress-associated apoptosis involves pathways like CHOP activation, IRE1-mediated JNK signaling, and altered calcium homeostasis. ER stress also exacerbates liver inflammation through activation of inflammatory pathways like NF-κB, JNK, and IKK. Importantly, ER stress has been linked to the development of HCC in MAFLD, suggesting its role in malignant transformation [24].

Mitochondria, key ROS producers, are susceptible to damage, exacerbating ROS production and affecting ATP production [24]. In MAFLD, mitochondrial ROS production is heightened due to reduced glutathione levels, impaired mitochondrial respiratory chain, and increased cytochrome P450 2E1 activity [24]. This contributes to liver injury and steatosis. Kupffer cells and hepatic stellate cells are also affected by oxidative stress, leading to inflammation and fibrosis. The resultant inflammatory response further damages hepatocytes, promoting fibrotic cell proliferation and potentially fostering liver tumor growth.

Compounds like luteolin, tomatidine, oxymatrine, and oleanolic acid regulate glucose homeostasis and lipid synthesis by decreasing the expression of key adipogenesis-related genes [24]. Betaine activates AMPK and downregulates SREBP-1c, thereby improving hepatic steatosis, while nuciferine, baicalein, puerarin, and emodin also exhibit promising effects in mitigating lipid accumulation through various mechanisms such as inhibiting adipogenic transcription factors or enhancing fatty acid oxidation [24]. Additionally, compounds like nordihydroguaiaretic acid and schizandrin A may reduce obesity by increasing fatty acid oxidation, presenting potential therapeutic avenues for conditions like MAFLD and MASH [24].

Natural products with anti-inflammatory properties hold promise for treating MASH. Compounds like resveratrol, celastrol, nuciferine, and emodin exhibit anti-inflammatory effects by targeting various pathways such as NF-κB and TLR4 signaling [24]. Isoorientin, geraniol, astaxanthin, schisandrin B, kukoamine B, genistein, naringenin, and scopolamine also demonstrate anti-inflammatory properties by modulating cytokine levels and oxidative stress markers in animal models of MASH [24]. These findings underscore the potential of natural compounds as therapeutic agents against MASH-related inflammation and its associated complications.

Liver fibrosis is a critical factor in the progression of MASH and significantly impacts patient outcomes, often leading to cirrhosis and other severe liver diseases. Excessive fibrosis, primarily regulated by hepatic stellate cells (HSCs), signifies advanced disease stages and increases the risk of hepatocellular carcinoma (HCC) and mortality [24]. Preventing and controlling liver fibrosis are thus crucial in managing MASH progression. Various natural compounds, such as isorhamnetin, astragaloside, salvianolic acid B, pycnogenol, calycosin, glycyrrhetinic acid, xanthohumol, thymoquinone, and isochlorogenic acid B, exhibit promising anti-fibrotic effects by targeting oxidative stress, inflammation, and fibrosis-related pathways [24]. These compounds hold potential as therapeutic interventions to attenuate liver fibrosis and mitigate the progression of MASH-related liver diseases.

### 3.2. Risk Factors

MASH, a widely acknowledged contributor to cirrhosis, is progressively linked to the onset of HCC. MASH, a clinical syndrome sharing pathological features with alcoholic hepatitis but without significant alcohol consumption, represents a distinct manifestation of metabolic-associated fatty liver disease (MAFLD). Marked by hepatocellular inflammation related to steatosis, MASH, if aggravated, can advance to cirrhosis, potentially culminating in HCC. HCC in the context of MASH is linked to various risk factors, including advanced liver fibrosis, older age, male gender, and the presence of metabolic syndrome. Genetic factors and dietary patterns are additional factors associated with the development of MASH-related HCC. Consequently, a systematic approach to HCC surveillance, akin to protocols for chronic liver diseases from other causes, may be necessary for individuals with MAFLD. It is important to highlight that the identification of MASH-related HCC demands the creation of rapid, specific, and straightforward diagnostic markers. Thus, the key challenge lies in identifying the risk factors for HCC development in MAFLD patients and implementing cost-effective strategies for screening and diagnosis. The risk factors for MASH can be classified as either genetic or non-genetic [25].

#### 3.2.1. Genetic Risk Factors

Extensive research has established a connection between genetic polymorphisms and the onset of MAFLD and MASH. However, few studies have investigated the genetic factors associated with MAFLD-related HCC. Initial investigations into MAFLD revealed variations across ethnic groups in terms of disease prevalence, with Hispanics exhibiting the highest incidence, followed by Caucasians and African Americans [26,27,28]. In the past few years, extensive genome-wide investigations have significantly advanced our understanding of MAFLD and MASH, potentially shedding light on the risk of developing HCC. Romeo and colleagues [29] launched the first genome-wide association study in the Dallas Heart Study. The only genetic variation identified with a strong correlation to hepatic steatosis was Patatin-like phospholipase domain 3, PNPLA3 (rs738409). Despite the unclear mechanism through which PNPLA3 contributes to the accumulation of hepatic steatosis, it has been demonstrated to be involved in the remodeling of lipid droplets within hepatocytes and the secretion of very low-density lipoproteins [30,31]. In subsequent investigations, the transmembrane 6 superfamily member 2, TM6SF2 (rs58542926), emerged as another significant finding and was recognized in an exome-wide association study focusing on fatty liver and serum aminotransferases [32]. While the connection between TM6SF2 and MAFLD is firmly established, debates persist regarding its association with the development of HCC [33].

#### 3.2.2. Non-Genetic Risk Factors

##### Diabetes

Diabetes plays a detrimental role in individuals with liver diseases, mainly contributing to the progression of cirrhosis in MASH patients and elevating the likelihood of liver cancer in those with MASH and MASH-related cirrhosis [34]. Observational research indicates a substantial elevation in the risk of developing HCC, ranging from two to four times, in individuals with type 2 diabetes. Several studies have considered possible confounding factors, including alcohol consumption and viral hepatitis, when investigating the link between diabetes and HCC. The influential VA study led by El-Serag et al. [35] revealed, over a 10-year follow-up of 173,643 veterans, a substantial increase in the risk of HCC associated with type 2 diabetes (T2D). Subsequent studies not only confirmed these findings [36,37] but also indicated a heightened HCC risk with an increasing number of metabolic syndrome (MetS) features [37]. In a prospectively collected cohort from the Nurses’ Health Study and Health Professionals’ Health Study, Simon et al. [37] reported adjusted hazard ratios (HRs) for HCC in diabetes patients at 5.8 (95% CI: 3.49–9.64) for women and 5.49 (95% CI: 3.16–9.51) for men, compared to their non-diabetic counterparts, after accounting for baseline characteristics. In a retrospective study involving 6508 Japanese individuals diagnosed with MAFLD through ultrasonography and a median follow-up of 5.6 years, 16 new cases of HCC (0.25%) were identified. The multivariate analysis highlighted diabetes (HR: 3.21; 95% CI: 1.09–9.50; *p* = 0.035), serum AST level ≥ 40 IU/L (HR: 8.20; 95% CI: 2.56–26.26; *p* < 0.001), platelet count < 150 × 10^3^/μL (HR: 7.19; 95% CI: 2.26–23.26; *p* = 0.001), and age ≥ 60 years (HR: 4.27; 95% CI: 1.30–14.01; *p* = 0.017) as independent risk factors for HCC [38]. Patients with good glycemic control (defined as HbA1c < 7% for >80% time) were associated with a 32% lower risk of HCC than patients who had suboptimal glycemic control (HR, 0.68; 95% CI, 0.60–0.77; *p* < 0.0001). Patients with diabetes complications had a 24% higher risk of HCC than patients without diabetes complications (HR, 1.24; 95% CI, 1.12–1.38; *p* < 0.0001) [39].

##### Obesity

There are several reasons why obesity poses a risk for HCC. Evidence indicates that obesity is associated with insulin resistance and elevated insulin-like growth factor, which triggers cell growth as a mitogen. A meta-analysis established that being overweight independently contributes to the risk of liver cancer. Among the eleven cohort studies considered, seven involved overweight individuals (n = 5037), and ten included obese individuals (n = 6042) [40]. In comparison to those with normal weight, the relative risks for HCC were 1.17 (95% CI: 1.02–1.34) for those with overweight and 1.89 (95% CI: 1.51–2.36) for those classified as obese [40]. In a distinct study involving 25,337 individuals diagnosed with hepatocellular carcinoma (HCC) in 26 prospective studies, overweight and obesity were linked to an 18% and 83% heightened risk of HCC, respectively. This association was consistent regardless of gender and geographical location. While the incidence appeared to be greater in men than in women, this contrast might be explained by differences in the distribution of adipose tissue, particularly a higher prevalence of visceral obesity in men [41]. Certain studies have utilized BMI as a criterion for diagnosing obesity, overlooking cirrhosis and disregarding the presence of ascites. In these investigations, potential confounding factors such as advanced chronic liver disease and obesity should be considered and controlled for during analysis, as biases may arise. Another study examined 19,271 patients, with an overall hepatocellular carcinoma incidence of 3.4% (n = 659). Obesity emerged as an independent predictor for liver cancer in individuals with alcoholic cirrhosis (OR 3.2; 95% CI, 1.5–6.6; *p* = 0.002) and cryptogenic cirrhosis (OR, 11.1; 95% CI, 1.5–87.4; *p* = 0.02) [42]. Notably, it has been observed that some individuals with cryptogenic cirrhosis have MAFLD as an underlying etiology [43].

##### Body Mass Index (BMI)

BMI has traditionally been a measure of obesity in epidemiological studies. While easily accessible in clinical settings, BMI lacks information on adipose distribution, particularly distinguishing between visceral and peripheral fat, each carrying distinct metabolic health implications. Early studies in cirrhotic patients revealed a higher risk of mortality in those with visceral adiposity compared to those with peripheral adipose tissue. Ioannou et al. [44] skillfully demonstrated these associations using the National Health and Nutritional Examination Survey, categorizing patients based on central or peripheral adipose distribution. Among those with central adipose distribution, individuals in the obese group (BMI ≥ 30 kg/m^2^) exhibited higher rates of cirrhosis-related death and hospitalizations (adjusted HR = 2.2, 95% CI: 1.1–4.6) compared to normal-weight counterparts (BMI < 25 kg/m^2^), a trend not observed in those with increased peripheral adipose distribution. In the context of MAFLD and MAFLD-associated HCC, central obesity, a prominent feature of metabolic syndrome (MetS), provides more meaningful insights into metabolic health [45,46].

##### Hypertension

The evidence regarding hypertension, a component often included in various definitions of metabolic syndrome (MetS), is inconclusive, with some studies identifying it as a risk factor and others not [47,48]. Additionally, many studies assess the combined features of MetS to gauge associated risks. Consequently, the specific impact of hypertension in isolation, without the presence of other MetS features, remains uncertain. Numerous epidemiological investigations have identified a bidirectional and reciprocal relationship between hypertension (HTN) and metabolic-associated fatty liver disease (MAFLD), indicating that the likelihood of developing MAFLD is elevated in individuals with HTN and vice versa [49,50]. In a meta-analysis conducted by Ciardullo et al. [51], which encompassed 11 longitudinal studies, it was revealed that individuals with MAFLD faced a 66% increased risk of developing HTN (HR: 1.66, CI: 1.38–2.01), with variations in prevalence associated with age and BMI of the patients. Patients with hypertension also exhibited an increased prevalence of advanced fibrosis, ranging from 3% to 9%, depending on the specific biomarker employed [51]. Ciardullo et al. [51] employed information from the National Health and Nutrition Examination Survey in the 2017–2018 cycle to conduct a cross-sectional examination. The findings revealed a gradual increase in the risk of steatosis associated with blood pressure.

##### Hyperlipidemia

Dyslipidemia stands out as a critical risk factor for cardiovascular diseases, intimately linked to both metabolic syndrome and obesity [52]. Liver cells are notably impacted by the ectopic accumulation of lipids, considering the liver’s pivotal role in regulating systemic lipid and glucose levels. Fatty liver is intricately associated with dyslipidemia and dysglycemia, independently of visceral fat presence [53]. Consequently, MAFLD and MASH emerge as prevalent liver disorders in the context of dyslipidemia, exhibiting strong connections with insulin resistance, an increased risk of progressing to liver cirrhosis, and the potential development of HCC [54]. Within the tumor microenvironment, adipocytes assume a vital role through the secretion of various molecular mediators. Adipose tissue releases adipokines such as leptin, adiponectin, resistin, and inflammatory mediators like ANGPTL2. These compounds regulate insulin sensitivity and trigger persistent low-grade inflammation. The imbalanced release of adipokines by adipocytes plays a substantial role in the emergence of metabolic disorders linked to obesity [55].

##### Obstructive Sleep Apnea (OSA)

OSA has become a noteworthy consideration, particularly in the current discussion on whether OSA is an independent factor that contributes to the development of MASH. While a limited number of previous trials have identified OSA as a risk factor for MASH, there are contrasting viewpoints, with some studies characterizing this association as coincidental rather than a substantial correlation. Another study proposed that severe OSA could potentially be a risk factor for MASH, independent of the patient’s body weight [56,57,58,59].

A notable discovery from these studies emphasizes the link between Obstructive Sleep Apnea (OSA) and metabolic-associated fatty liver disease (MAFLD), particularly concerning the degree of nocturnal hypoxemia associated with OSA. Animal models have primarily concentrated on intermittent hypoxia, a key characteristic of OSA, to uncover how OSA might play a role in the complex metabolic disruptions observed in MAFLD. Intermittent hypoxia induces tissue hypoxia and can lead to oxidative stress, mitochondrial dysfunction, inflammation, and heightened activation of the sympathetic nervous system, among other maladaptive effects. In these models, intermittent hypoxia has been demonstrated to induce insulin resistance, impair key steps in hepatic lipid metabolism, promote atherosclerosis, and contribute to hepatic steatosis and fibrosis, all of which are relevant to MAFLD initiation and/or progression [60].

##### Cardiovascular Disease

Clinical presentations linked to metabolic-associated fatty liver disease (MAFLD), including steatosis and inflammation, represent supplementary elements that contribute to the susceptibility to cardiovascular disease (CVD) [61]. Those experiencing new CVD events had a significantly higher estimated 10-year CVD risk (17% vs. 10%) as determined by the Framingham risk score (FRS) compared to MAFLD patients without such events [62]. Over an 8-year follow-up period, the mortality rate among patients with MAFLD was higher than that observed in the general population. In another study involving biopsy-diagnosed MAFLD patients followed for 18 years, CVD emerged as one of the leading causes of death, surpassing the collective mortality from all types of cancers combined [63]. Individuals with metabolic-associated fatty liver disease (MAFLD) exhibit elevated occurrences of clinical coronary artery disease (CAD) and experience poorer outcomes following coronary events. In a prospective investigation conducted by Patel et al., which involved 228 patients undergoing coronary angiography as part of a liver transplant assessment, individuals diagnosed with metabolic dysfunction-associated steatohepatitis (MASH) exhibited notably higher rates of severe coronary artery disease (CAD) after adjusting for conventional CAD risk factors. This was in comparison to individuals with hepatitis C or alcohol-related cirrhosis. Moreover, patients with metabolic-associated fatty liver disease (MAFLD) demonstrated an increased prevalence of coronary lesions requiring percutaneous coronary intervention, heightened in-hospital mortality during episodes of acute coronary syndrome, and elevated 3-year mortality following acute ST-segment elevation myocardial infarction [64,65].

##### Cerebrovascular Accidents

Metabolic-associated fatty liver disease (MAFLD) seems to elevate the occurrence of ischemic stroke, although conflicting evidence exists regarding its potential role as a causative factor. Earlier, smaller studies did not present a definitive association between these two conditions [66,67]. Distinct ischemic stroke patterns associated with metabolic-associated fatty liver disease (MAFLD) have been under scrutiny. Among stroke patients with MAFLD, occurrences of large artery atherosclerosis and small vessel occlusions are more prevalent, whereas a cardioembolic origin is less frequently identified [68]. Additionally, this patient population may experience a higher incidence of brainstem infarctions, with an elevated risk of progression even after adjusting for comorbidities [69]. Elevated levels of aminotransferases and gamma-glutamyl transferase (gGT), primarily linked to metabolic-associated fatty liver disease (MAFLD), have been shown to be associated with an increased occurrence of ischemic stroke in several investigations. In a case–control study involving 103 individuals with acute ischemic stroke and 200 controls, both alanine and aspartate aminotransferase levels were independently associated with an elevated odds ratio for ischemic stroke. [70]. In a larger prospective study with 6997 men without established cardiovascular disease (CVD) or type 2 diabetes mellitus (T2DM), gGT levels, a more specific marker of MAFLD, were independently associated with a higher risk of ischemic stroke, even among individuals at low or moderate cardiovascular risk [71]. In the EUROSTROKE study, a nested case–control study carried out in three European countries (Finland, the Netherlands, and the United Kingdom), the correlation between gGT levels and the likelihood of ischemic stroke seemed more prominent in individuals without type 2 diabetes mellitus (T2DM) [72]. Significantly, gGT appears to contribute to atherogenesis [73], as it has been identified in atheromatic plaques, macrophages, and foam cells [74]. Its role in atherosclerosis is proposed to involve the induction of oxidative stress [75]. individuals with MAFLD fibrosis, identified through the FIB-4 index, showed higher rates of stroke compared to those without fibrosis, according to data from the United States National Health and Nutrition Examination Survey (NHANES) spanning from 2005 to 2014 (odds ratio 1.87, 95% confidence interval 1.00–3.50) [76].

##### Chronic Kidney Disease

Patients with MASH exhibit a higher incidence of chronic kidney disease (CKD) compared to other causes, leading to MASH emerging not only as a primary indication for LT [51,77] but also for simultaneous liver–kidney transplantation (SLKT) in the United States. This is attributed to the significance of serum creatinine and dialysis status in the model for end-stage liver disease (MELD) score [78]. With the rising incidence of renal dysfunction at LT due to prioritization under the MELD allocation system in the United States, the rates of SLKT have increased from 2.7% of all LT in 2000 to 9.3% in 2016.

The association between metabolic-associated fatty liver disease (MAFLD), particularly its necro-inflammatory form (MASH), and kidney disease remains not fully understood. The liver, a central controller of glucose and lipid metabolism, significantly impacts the emergence of cardiovascular and kidney diseases. Recent findings indicate that MAFLD, specifically MASH, may not just indicate kidney damage but might actively participate in its onset. Possible mechanisms include the release of pathogenic mediators from the inflamed liver, such as reactive oxygen species, advanced glycation end products, and inflammatory molecules. Pro-inflammatory and pro-fibrogenic substances released by the liver may promote kidney injury. The presence of hepatorenal syndrome in cirrhotic patients further supports the interconnected pathways between the liver and kidneys. Although evidence links MAFLD to chronic kidney disease (CKD), a definitive causal relationship has not been conclusively established. MAFLD may exacerbate insulin resistance, contribute to atherogenic dyslipidemia, and release pathogenic mediators that play a role in CKD pathophysiology [79,80].

##### Alcohol Consumption

Several epidemiological studies suggest a protective influence of light to moderate daily alcohol consumption against the development of metabolic-associated fatty liver disease (MAFLD). However, while these modest ethanol amounts may deter fatty liver, they might pose a risk for other conditions like breast and colon cancer. Individuals with underlying hepatic steatosis or metabolic dysfunction-associated steatohepatitis (MASH) are advised against chronic ethanol use, as current data do not endorse a favorable impact of alcohol in such cases. Exceptionally, overweight and obese individuals may be more vulnerable to the effects of alcohol, even at moderate levels [81]. The analysis of the relationship between alcohol consumption and the occurrence of HCC and mortality from liver disease revealed a significant association [82]. Specifically, consumption of more than three alcoholic drinks daily was significantly linked to both the incidence of HCC (HR: 1.92; 95% CI: 1.42–2.60) and mortality from liver disease (HR: 5.84; 95% CI: 4.81–7.10), compared to those consuming up to one drink per day [82]. An approximate intake of more than 80 g alcohol per day leads to the RR ranging between 4.5 and 7.3 for hepatocellular carcinoma (HCC), compared with abstinence or consumption of less than 40 g per day [83]. In metabolic-associated fatty liver disease (MAFLD), the accumulation of fat in hepatocytes is primarily driven by the metabolic syndrome, marked by hyperinsulinemia and elevated levels of circulating free fatty acids [84,85]. This set of conditions is marked by obesity, diabetes mellitus (DM), hypertension, and disturbances in fat metabolism. Notably, moderate alcohol consumption has been shown to positively affect peripheral insulin resistance, providing benefits for individuals with type II DM [86].

##### Smoking

Smoking has been linked to a higher risk of developing HCC [87,88], although there have not been specific studies exploring the connection between smoking and HCC related to MAFLD. The liver metabolizes tobacco carcinogens, and the creation of DNA adducts could serve as a crucial factor initiating hepatocarcinogenesis [89].

##### Gut Microbiome

In recent years, there has been an increased understanding that the microbiota, a diverse ecosystem comprising bacteria, archaea, protists, fungi, and viruses residing in the human gut, is not a passive observer but an active participant in human physiology. Various factors, including host-related aspects such as diet, physical activity, medication, circadian rhythm, and geographical location, influence the composition and function of the microbiota. This intricate community of microorganisms possesses a significantly greater amount of genetic information compared to the human genome. For instance, it contains enzymes capable of biochemical functions absent in the human host, such as deconjugating primary bile acids or the breakdown of indigestible carbohydrates. The unique configuration of an individual’s microbiota collaborates with their specific genetic makeup, contributing to personalized traits and phenotypes.

Several possible mechanisms through which the gut microbiota influences MAFLD and MASH have been explored in recent studies. Proposed mechanisms involve dysbiotic bacteria and their byproducts moving to the liver due to a compromised gut barrier. This migration triggers an inflammatory response in the liver, and there are also interactions between commensal microbes or metabolites and dietary factors that contribute to the development of steatosis [90].

Patients with MAFLD and especially MASH have been shown to exhibit an increased number of Bacteroidetes and differences in the presence of Firmicutes [91]. Apart from this difference, patients with MAFLD have also been demonstrated to exhibit an increased proportion of species belonging to Clostridium, Anaerobacter, Streptococcus, Escherichia, and Lactobacillus, whereas Oscillibacter, Flavonifaractor, Odoribacter, and Alistipes spp. are less prominent [92]. Furthermore, there is a relative abundance of potential pathogens, such as Gram-negative Proteobacteria, Enterobacteriaceae, and Escherichia spp. among patients with MASH, when compared to healthy controls, while Faecalibacterium prausnitzii, and Akkermansia muciniphila are relatively diminished [93,94]. Changes in gut microbiota are associated with increased fecal concentrations of 2-butanone and 4-methyl-2-pentanone, metabolites that can induce liver cell toxicity in individuals with metabolic liver diseases, as compared to healthy individuals [95]. Furthermore, the gut microbiota in patients with MAFLD contains a higher concentration of ethanol-producing bacteria, such as *E. coli*, which can produce ethanol without oxygen [96]. This leads to a suggestion that the gut microbiota in these patients may generate more ethanol than that of healthy individuals, as indicated by higher levels of intrinsically produced ethanol in the bloodstream and breath. Ethanol is known to trigger Nuclear-Factor-kappa-B signaling molecules, causing tissue damage by impairing gut barrier function and thereby increasing portal lipopolysaccharide concentrations. It has been noted that the detoxification process is compromised in the liver of patients with MAFLD, leading to a rise in the production of reactive oxygen species (ROS) [96]. These ROS can cause oxidative damage to liver cells, increasing liver inflammation and contributing to MASH [96].

##### Iron Overload

Patients with HCC have been noted to exhibit iron overload, and hepatic iron overload associated with MAFLD could contribute to carcinogenesis through oxidative stress. Additionally, elevated serum ferritin levels, indicative of hyperferritinemia, might be a factor that increases the risk of liver fibrosis progression and HCC in MASH. As a result, individuals with heightened serum ferritin levels may require screening for HCC. While there is evidence suggesting iron overload as a risk factor for HCC, it remains unclear whether this condition is a cause or consequence of advanced liver disease [97,98]. Hyperferritinemia, specifically in patients with HFE hemochromatosis, is associated with a high risk of developing hepatocellular carcinoma [99]. In these patients, the morbidity and mortality of patients in whom phlebotomy is initiated before the development of cirrhosis and diabetes are significantly reduced [99]. However, in patients without HFE hemochromatosis and solely MAFLD, the data are limited. Phlebotomy has been observed in studies to significantly decrease insulin resistance and increase levels of alanine transaminase and triglycerides [100]. While phlebotomy might be an effective way to reduce iron storage in the liver of MAFLD patients, there is no significant correlation between serum ferritin levels and inflammation or erythrocyte sedimentation rate [101]. Additionally, serum ferritin levels do not necessarily predict the severity of MAFLD.

#### 3.2.3. Clinical Features

MAFLD is frequently asymptomatic and is typically discovered incidentally during medical assessments, mainly through liver ultrasonography conducted for other purposes. Alternatively, it can be identified based on clinical indicators of the metabolic syndrome [102,103]. Consequently, it is understandable how cardiovascular complications, primarily arising from atherosclerosis, valvular calcifications, and heightened intimal arterial thickness, constitute the primary causes of mortality and morbidity in individuals with MAFLD [104,105]. Diagnosing metabolic-associated fatty liver disease-related hepatocellular carcinoma (MAFLD-HCC) often occurs at a more advanced stage when compared to HCC, stemming from other causes [106]. A sizable Italian cohort revealed that at the presentation of MAFLD-HCC, Barcelona Clinic Liver Cancer (BCLC) C tumors were significantly more prevalent than those in HCV-HCC cases (21% vs. 4%, *p* < 0.0001) [47]. Both insufficient HCC surveillance practices and the occurrence of MAFLD-HCC in the absence of cirrhosis, leading to the absence of surveillance practices, are likely contributing factors.

#### 3.2.4. Pharmacological Therapies

Metformin is a medication commonly used to manage type 2 diabetes mellitus (T2DM). It has been linked to a lower risk of cirrhosis and hepatocellular carcinoma (HCC) in T2DM patients with chronic liver disease. Metformin may also inhibit cancer invasion and metastasis, potentially improving patient outcomes, but more research is needed [107].

PPARα agonists, like glitazones, have shown beneficial effects in patients with MAFLD and MASH. Pioglitazone, for instance, has been found to improve liver function and liver fat content, and can lead to the resolution of MASH in patients, regardless of their T2DM status. However, its effect on liver fibrosis is modest. Rosiglitazone, on the other hand, has shown limited effects and its trials were discontinued due to increased cardiovascular risk [107]. Despite these promising results, the use of pioglitazone for MASH is currently not approved beyond the treatment of T2DM due to potential side effects like weight gain, fluid retention, and risk of bone fractures or bladder cancer. However, pioglitazone has been found to lower the risk of myocardial infarction and stroke in patients with T2DM or prediabetes, making it a potential treatment for MASH patients at risk of cardiovascular disease [107]. Other agents, like PPARα/δ and PPARα/γ agonists, are currently being studied for their ability to safely metabolize substrates. Elafibranor, a PPARα/δ agonist, improves insulin resistance and inflammation, and has shown promise in early clinical trials. Saroglitazar, a PPARα/γ dual agonist, has shown potential benefits in animal models of MASH and may decrease serum ALT concentrations and improve cardiometabolic profiles. However, larger clinical trials are needed to confirm these results [107]. Sodium-glucose cotransporter-2 (SGLT-2) inhibitors have been found to have a positive effect on liver steatosis, inflammation, and fibrosis, making them a potential treatment for MASH. However, most of the randomized controlled trials (RCTs) conducted so far have been small and have not tested the impact of SGLT-2 inhibitors on liver histology [108].

Statins and other lipid-lowering agents are often used to manage conditions associated with MAFLD, such as type 2 diabetes mellitus (T2DM), hypertension, obesity, and dyslipidemia [108]. Statins inhibit a key enzyme in cholesterol synthesis and have been shown to reduce cardiovascular morbidity in MAFLD patients without causing significant liver damage [108]. They may also improve liver steatosis, inflammation, and fibrosis. Other lipid-lowering agents, such as ezetimibe, fenofibrate, and omega-3 polyunsaturated fatty acids, have varying effects on liver histology in MAFLD patients [108].

Inhibitors of angiotensin converting enzyme (ACEi) or angiotensin II receptor blockers (ARBs) may have anti-fibrotic effects on the liver, but results from clinical studies have been inconsistent [108]. Anti-platelet aggregation agents, like aspirin, have been associated with less severe histological features of MAFLD and MASH and a lower risk of progression to advanced fibrosis [108]. Vitamin E, a potent antioxidant, has shown potential in the treatment of MAFLD in preclinical studies and in a trial involving non-diabetic patients with MASH [108].

Several new drugs are being tested for MAFLD, including synthetic ligands that activate the farnesoid X receptor (FXR), thyroid hormone receptor (THR)-β-selective agonists, inhibitors of Diacylglycerol-O-acyltransferase 2 (DGAT2), and agents that modulate the immune system or inhibit apoptosis [108]. However, results from clinical trials have been mixed, as seen for a few interventions in Table 1, and more research is needed to determine the effectiveness and safety of these agents.

## 4. Conclusions

MAFLD is often asymptomatic and is typically detected incidentally during medical examinations, often through liver ultrasonography conducted for other reasons or based on clinical signs of metabolic syndrome. However, the prevalence of the condition is on the rise. Diagnosis of MAFLD-related hepatocellular carcinoma (MAFLD-HCC) frequently occurs at a more advanced stage compared to HCC from other causes. Symptoms of MASH-related HCC may include fatigue, abdominal pain, weight loss, jaundice, swelling, and easy bruising. Insufficient HCC surveillance and the occurrence of MAFLD-HCC without cirrhosis, resulting in a lack of surveillance, are likely contributing factors. Additionally, understanding the pathogenesis of the disease might provide profound treatment therapies that are targeted and might aid in reducing the development of HCC.

## Figures and Tables

**Table 1:** Recent trials and systematic review about treatment approaches.

Author	Study	Participants	Objectives	Outcomes
Ramai et al. [109]	Systematic Review and Meta-analysis	19,514,750 patients (18,423,546 controls and 1,091,204 bariatric patients)	To determine whether bariatric surgery reduces the risk of HCC.	The pooled rate/1000 person-years was 0.05 (95% CI: 0.02–0.07) in bariatric surgery patients and 0.34 (95% CI: 0.20–0.49) in the control group with an incidence rate ratio of 0.28 (95% CI: 0.18–0.42).
Harrison et al. [110]	Randomized Controlled Trial	38 participants divided to placebo or treatment group	To assess the performance of a structurally optimized FXR agonist in patients with MASH.	MET409 lowered LFC over 12 weeks in patients with MASH and delivered a differentiated pruritus and LDL-C profile at 50 mg, providing the first clinical evidence that the risk–benefit profile of FXR agonists can be enhanced through structural optimization.
Jiang et al. [111]	Randomized Controlled Trial	48 participants 30 received MET409 and 18 received a placebo.	To investigate potential early predictors of the 12-week treatment response with MET409	The relative change in the MRI-based proton density fat fraction (PDFF) at week 4 was highly predictive of the treatment response estimated by using the week 12 MRI-based PDFF.

FXR: farnesoid X receptor, LFC: liver fat content.

## Data Availability

Data can be made available on special request addressed to the corresponding author.

## References

[1] Teng M.L., Ng C.H., Huang D.Q., Chan K.E., Tan D.J., Lim W.H., Yang J.D., Tan E., Muthiah M.D. (2023). Global incidence and prevalence of nonalcoholic fatty liver disease. Clin. Mol. Hepatol..

[2] Le M.H., Le D.M., Baez T.C., Wu Y., Ito T., Lee E.Y., Lee K., Stave C.D., Henry L., Barnett S.D. (2023). Global incidence of non-alcoholic fatty liver disease: A systematic review and meta-analysis of 63 studies and 1,201,807 persons. J. Hepatol..

[3] Riazi K., Azhari H., Charette J.H., Underwood F.E., King J.A., Afshar E.E., Swain M.G., Congly S.E., Kaplan G.G., Shaheen A.A. (2023). The prevalence and incidence of NAFLD worldwide: A systematic review and meta-analysis. Lancet Gastroenterol. Hepatol..

[4] Li J., Zou B., Yeo Y.H., Feng Y., Xie X., Lee D.H., Fujii H., Wu Y., Kam L.Y., Ji F. (2019). Prevalence, incidence, and outcome of non-alcoholic fatty liver disease in Asia, 1999–2019: A systematic review and meta-analysis. Lancet Gastroenterol. Hepatol..

[5] Cholongitas E., Pavlopoulou I., Papatheodoridi M., Markakis G.E., Bouras E., Haidich A.B., Papatheodoridis G. (2021). Epidemiology of nonalcoholic fatty liver disease in Europe: A systematic review and meta-analysis. Ann. Gastroenterol..

[6] Rojas Y.A.O., Cuellar C.L.V., Barrón K.M.A., Arab J.P., Miranda A.L. (2022). Non-alcoholic fatty liver disease prevalence in Latin America: A systematic review and meta-analysis. Ann. Hepatol..

[7] Younossi Z., Anstee Q.M., Marietti M., Hardy T., Henry L., Eslam M., George J., Bugianesi E. (2018). Global burden of NAFLD and NASH: Trends, predictions, risk factors and prevention. Nat. Rev. Gastroenterol. Hepatol..

[8] Zhang X., Heredia N.I., Balakrishnan M., Thrift A.P. (2021). Prevalence and factors associated with NAFLD detected by vibration controlled transient elastography among US adults: Results from NHANES 2017–2018. PLoS ONE.

[9] Younossi Z.M., Golabi P., Paik J.M., Henry A., Van Dongen C., Henry L. (2023). The global epidemiology of nonalcoholic fatty liver disease (NAFLD) and nonalcoholic steatohepatitis (NASH): A systematic review. Hepatology.

[10] Loomba R., Lim J.K., Patton H., El-Serag H.B. (2023). AGA Clinical Practice Update on Screening and Surveillance for Hepatocellular Carcinoma in Patients with Nonalcoholic Fatty Liver Disease: Expert Review. Gastroenterology.

[11] Foerster F., Gairing S.J., Müller L., Galle P.R. (2022). NAFLD-driven HCC: Safety and efficacy of current and emerging treatment options. J. Hepatol..

[12] Pinyopornpanish K., Khoudari G., Saleh M.A., Angkurawaranon C., Pinyopornpanish K., Mansoor E., Dasarathy S., McCullough A. (2021). Hepatocellular carcinoma in nonalcoholic fatty liver disease with or without cirrhosis: A population-based study. BMC Gastroenterol..

[13] Younossi Z.M., Koenig A.B., Abdelatif D., Fazel Y., Henry L., Wymer M. (2016). Global epidemiology of nonalcoholic fatty liver disease-Meta-analytic assessment of prevalence, incidence, and outcomes. Hepatology.

[14] Estes C., Anstee Q.M., Arias-Loste M.T., Bantel H., Bellentani S., Caballeria J., Colombo M., Craxi A., Crespo J., Day C.P. (2018). Modeling NAFLD disease burden in China, France, Germany, Italy, Japan, Spain, United Kingdom, and United States for the period 2016–2030. J. Hepatol..

[15] Donnelly K.L., Smith C.I., Schwarzenberg S.J., Jessurun J., Boldt M.D., Parks E.J. (2005). Sources of fatty acids stored in liver and secreted via lipoproteins in patients with nonalcoholic fatty liver disease. J. Clin. Investig..

[16] Samuel V.T., Shulman G.I. (2012). Mechanisms for insulin resistance: Common threads and missing links. Cell.

[17] Caligiuri A., Gentilini A., Marra F. (2006). Molecular Pathogenesis of NASH. Int. J. Mol. Sci..

[18] Prisco M., Romano G., Peruzzi F., Valentinis B., Baserga R. (1999). Insulin and IGF-I receptors signaling in protection from apoptosis. Horm. Metab. Res..

[19] Kaji K., Yoshiji H., Kitade M., Ikenaka Y., Noguchi R., Yoshii J., Yanase K., Namisaki T., Yamazaki M., Moriya K. (2008). Impact of insulin resistance on the progression of chronic liver diseases. Int. J. Mol. Med..

[20] Neuschwander-Tetri B.A. (2010). Hepatic lipotoxicity and the pathogenesis of nonalcoholic steatohepatitis: The central role of nontriglyceride fatty acid metabolites. Hepatology.

[21] Perry R.J., Samuel V.T., Petersen K.F., Shulman G.I. (2014). The role of hepatic lipids in hepatic insulin resistance and type 2 diabetes. Nature.

[22] Terpstra V., van Amersfoort E.S., van Velzen A.G., Kuiper J., van Berkel T.J. (2000). Hepatic and extrahepatic scavenger receptors: Function in relation to disease. Arterioscler. Thromb. Vasc. Biol..

[23] Shi H., Kokoeva M.V., Inouye K., Tzameli I., Yin H., Flier J.S. (2006). TLR4 links innate immunity and fatty acid-induced insulin resistance. J. Clin. Investig..

[24] Shao G., Liu Y., Lu L., Zhang G., Zhou W., Wu T., Wang L., Xu H., Ji G. (2022). The Pathogenesis of HCC Driven by NASH and the Preventive and Therapeutic Effects of Natural Products. Front. Pharmacol..

[25] Gao J., Xie L., Yang W.S., Zhang W., Gao S., Wang J., Xiang Y.B. (2012). Risk factors of hepatocellular carcinoma--current status and perspectives. Asian. Pac. J. Cancer. Prev..

[26] Browning J.D., Szczepaniak L.S., Dobbins R., Nuremberg P., Horton J.D., Cohen J.C., Grundy S.M., Hobbs H.H. (2004). Prevalence of hepatic steatosis in an urban population in the United States: Impact of ethnicity. Hepatology.

[27] Caldwell S.H., Harris D.M., Patrie J.T., Hespenheide E.E. (2002). Is NASH underdiagnosed among African Americans?. Am. J. Gastroenterol..

[28] Browning J.D., Kumar K.S., Saboorian M.H., Thiele D.L. (2004). Ethnic differences in the prevalence of cryptogenic cirrhosis. Am. J. Gastroenterol..

[29] Romeo S., Kozlitina J., Xing C., Pertsemlidis A., Cox D., Pennacchio L.A., Boerwinkle E., Cohen J.C., Hobbs H.H. (2008). Genetic variation in PNPLA3 confers susceptibility to nonalcoholic fatty liver disease. Nat. Gen..

[30] Dongiovanni P., Donati B., Fares R., Lombardi R., Mancina R.M., Romeo S., Valenti L. (2013). PNPLA3 I148M polymorphism and progressive liver disease. World J. Gastroenterol..

[31] Smagris E., BasuRay S., Li J., Huang Y., Lai K.M., Gromada J., Cohen J.C., Hobbs H.H. (2015). Pnpla3I148M knockin mice accumulate PNPLA3 on lipid droplets and develop hepatic steatosis. Hepatology.

[32] Kozlitina J., Smagris E., Stender S., Nordestgaard B.G., Zhou H.H., Tybjærg-Hansen A., Vogt T.F., Hobbs H.H., Cohen J.C. (2014). Exome-wide association study identifies a TM6SF2 variant that confers susceptibility to nonalcoholic fatty liver disease. Nat. Genet..

[33] Falleti E., Cussigh A., Cmet S., Fabris C., Toniutto P. (2016). PNPLA3 rs738409 and TM6SF2 rs58542926 variants increase the risk of hepatocellular carcinoma in alcoholic cirrhosis. Dig. Liver Dis..

[34] Chrysavgis L., Giannakodimos I., Diamantopoulou P., Cholongitas E. (2022). Non-alcoholic fatty liver disease and hepatocellular carcinoma: Clinical challenges of an intriguing link. World J. Gastroenterol..

[35] El-Serag H.B., Tran T., Everhart J.E. (2004). Diabetes increases the risk of chronic liver disease and hepatocellular carcinoma. Gastroenterology.

[36] Lai S.W., Chen P.C., Liao K.F., Muo C.H., Lin C.C., Muo C.H., Lin C.C., Sung F.C. (2012). Risk of hepatocellular carcinoma in diabetic patients and risk reduction associated with anti-diabetic therapy: A population-based cohort study. Am. J. Gastroenterol..

[37] Chen H.F., Chen P., Li C.Y. (2010). Risk of malignant neoplasms of liver and biliary tract in diabetic patients with different age and sex stratifications. Hepatology.

[38] Kawamura Y., Arase Y., Ikeda K., Seko Y., Imai N., Hosaka T., Kobayashi M., Saitoh S., Sezaki H., Akuta N. (2012). Large-scale long-term follow-up study of Japanese patients with non-alcoholic Fatty liver disease for the onset of hepatocellular carcinoma. Am. J. Gastroenterol..

[39] Kramer J.R., Natarajan Y., Dai J., Yu X., Li L., El-Serag H.B., Kanwal F. (2022). Effect of diabetes medications and glycemic control on risk of hepatocellular cancer in patients with nonalcoholic fatty liver disease. Hepatology.

[40] Chen C.L., Yang H.I., Yang W.S., Liu C.J., Chen P.J., You S.L., Wang L.Y., Sun C.A., Lu S.N., Chen D.S. (2008). Metabolic factors and risk of hepatocellular carcinoma by chronic hepatitis B/C infection: A follow-up study in Taiwan. Gastroenterology.

[41] Yang J., He J., Feng Y., Xiang M. (2023). Obesity contributes to hepatocellular carcinoma development via immunosuppressive microenvironment remodeling. Front. Immunol..

[42] Nair S., Mason A., Eason J., Loss G., Perrillo R.P. (2002). Is obesity an independent risk factor for hepatocellular carcinoma in cirrhosis?. Hepatology.

[43] Caldwell S.H., Oelsner D.H., Iezzoni J.C., Hespenheide E.E., Battle E.H., Driscoll C.J. (1999). Cryptogenic cirrhosis: Clinical characterization and risk factors for underlying disease. Hepatology.

[44] Ioannou G.N., Weiss N.S., Boyko E.J., Kowdley K.V., Kahn S.E., Carithers R.L., Tsai E.C., Dominitz J.A. (2005). Is central obesity associated with cirrhosis-related death or hospitalization? A population-based, cohort study. Clin. Gastroenterol. Hepatol..

[45] Schlesinger S., Aleksandrova K., Pischon T., Fedirko V., Jenab M., Trepo E., Boffetta P., Dahm C.C., Overvad K., Tjønneland A. (2013). Abdominal obesity, weight gain during adulthood and risk of liver and biliary tract cancer in a European cohort. Int. J. Cancer.

[46] Pang Q., Zhang J.Y., Song S.D., Qu K., Xu X.S., Liu S.S., Liu C. (2015). Central obesity and nonalcoholic fatty liver disease risk after adjusting for body mass index. World J. Gastroenterol..

[47] Welzel T.M., Graubard B.I., Zeuzem S., El-Serag H.B., Davila J.A., McGlynn K.A. (2011). Metabolic Syndrome Increases the Risk of Primary Liver Cancer in the United States: A Study in the SEER-Medicare Database. Hepatology.

[48] Borena W., Strohmaier S., Lukanova A., Bjørge T., Lindkvist B., Hallmans G., Edlinger M., Stocks T., Nagel G., Manjer J. (2011). Metabolic Risk Factors and Primary Liver Cancer in a Prospective Study of 578,700 Adults. Int. J. Cancer.

[49] Ioannou G.N., Green P., Kerr K.F., Berry K. (2019). Models Estimating Risk of Hepatocellular Carcinoma in Patients with Alcohol or NAFLD-Related Cirrhosis for Risk Stratification. J. Hepatol..

[50] Younossi Z., Stepanova M., Ong J.P., Jacobson I.M., Bugianesi E., Duseja A., Eguchi Y., Wong V.W., Negro F., Yilmaz Y. (2019). Nonalcoholic Steatohepatitis Is the Fastest Growing Cause of Hepatocellular Carcinoma in Liver Transplant Candidates. Clin. Gastroenterol. Hepatol..

[51] Wong R.J., Aguilar M., Cheung R., Perumpail R.B., Harrison S.A., Younossi Z.M., Ahmed A. (2015). Nonalcoholic Steatohepatitis Is the Second Leading Etiology of Liver Disease among Adults Awaiting Liver Transplantation in the United States. Gastroenterology.

[52] Nilsson P.M., Tuomilehto J., Ryden L. (2019). The metabolic syndrome—What is it and how should it be managed?. Eur. J. Prev. Cardiol..

[53] Speliotes E.K., Massaro J.M., Hoffmann U., Vasan R.S., Meigs J.B., Sahani D.V., Hirschhorn J.N., O’Donnell C.J., Fox C.S. (2010). Fatty liver is associated with dyslipidemia and dysglycemia independent of visceral fat: The Framingham Heart Study. Hepatology.

[54] Rajesh Y., Sarkar D. (2021). Association of Adipose Tissue and Adipokines with Development of Obesity-Induced Liver Cancer. Int. J. Mol. Sci..

[55] Unamuno X., Gómez-Ambrosi J., Rodríguez A., Becerril S., Frühbeck G., Catalán V. (2018). Adipokine dysregulation and adipose tissue inflammation in human obesity. Eur. J. Clin. Investig..

[56] Ulitsky A., Ananthakrishnan A.N., Komorowski R., Wallace J., Surapaneni S.N., Franco J., Saeian K., Gawrieh S. (2010). A Noninvasive Clinical Scoring Model Predicts Risk of Nonalcoholic Steatohepatitis in Morbidly Obese Patients. Obes. Surg..

[57] Jouët P., Sabaté J.-M., Maillard D., Msika S., Mechler C., Ledoux S., Harnois F., Coffin B. (2007). Relationship between Obstructive Sleep Apnea and Liver Abnormalities in Morbidly Obese Patients: A Prospective Study. Obes. Surg..

[58] Weingarten T.N., Mantilla C.B., Swain J.M., Kendrick M.L., Oberhansley J.M., Burcham R.J., Ribeiro T.C.R., Watt K.D., Schroeder D.R., Narr B.J. (2012). Nonalcoholic Steatohepatitis in Bariatric Patients with a Diagnosis of Obstructive Sleep Apnea. Obes. Facts.

[59] Daltro C., Cotrim H.P., Alves E., de Freitas L.A., Araújo L., Boente L., Leal R., Portugal T. (2010). Nonalcoholic Fatty Liver Disease Associated with Obstructive Sleep Apnea: Just a Coincidence?. Obes. Surg..

[60] Mesarwi O.A., Loomba R., Malhotra A. (2019). Obstructive Sleep Apnea, Hypoxia, and Nonalcoholic Fatty Liver Disease. Am. J. Respir. Crit. Care Med..

[61] Feitosa M.F., Reiner A.P., Wojczynski M.K., Graff M., North K.E., Carr J.J., Borecki I.B. (2013). Sex-Influenced Association of Nonalcoholic Fatty Liver Disease with Coronary Heart Disease. Atherosclerosis.

[62] Akın L., Kurtoglu S., Yikilmaz A., Kendirci M., Elmalı F., Mazicioglu M. (2012). Fatty Liver Is a Good Indicator of Subclinical Atherosclerosis Risk in Obese Children and Adolescents Regardless of Liver Enzyme Elevation. Acta. Paediatr..

[63] Huang Y., Bi Y., Xu M., Ma Z., Xu Y., Wang T., Li M., Liu Y., Lu J., Chen Y. (2012). Nonalcoholic Fatty Liver Disease Is Associated with Atherosclerosis in Middle-Aged and Elderly Chinese. Arterioscler. Thromb. Vasc. Biol..

[64] Patel S.S., Nabi E., Guzman L., Abbate A., Bhati C., Stravitz R.T., Reichman T., Matherly S.C., Driscoll C., Lee H. (2018). Coronary Artery Disease in Decompensated Patients Undergoing Liver Transplantation Evaluation. Liver Transpl..

[65] Wong V.W., Wong G.L., Yeung J.C., Fung C.Y., Chan J.K., Chang Z.H., Kwan C.T., Lam H., Limquiaco J., Chim A.M. (2015). Long-term Clinical Outcomes after Fatty Liver Screening in Patients Undergoing Coronary Angiogram: A Prospective Cohort Study. Hepatology.

[66] Wen X., Wang S., Taveira T.H., Akhlaghi F. (2021). Required Warfarin Dose and Time in Therapeutic Range in Patients with Diagnosed Nonalcoholic Fatty Liver Disease (NAFLD) or Nonalcoholic Steatohepatitis (NASH). PLoS ONE.

[67] Moshayedi H., Ahrabi R., Mardani A., Sadigetegad S., Farhudi M. (2014). Association between Non-Alcoholic Fatty Liver Disease and Ischemic Stroke. Iran. J. Neurol..

[68] Wu M., Zha M., Lv Q., Xie Y., Yuan K., Zhang X., Liu X. (2022). Non-alcoholic Fatty Liver Disease and Stroke: A Mendelian Randomization Study. Eur. J. Neurol..

[69] Li H., Hu B., Wei L., Zhou L., Zhang L., Lin Y., Qin B., Dai Y., Lu Z. (2018). Non-alcoholic Fatty Liver Disease Is Associated with Stroke Severity and Progression of Brainstem Infarctions. Eur. J. Neurol..

[70] Ying I., Saposnik G., Vermeulen M.J., Leung A., Ray J.G. (2011). Nonalcoholic Fatty Liver Disease and Acute Ischemic Stroke. Epidemiology.

[71] Wannamethee S.G., Lennon L., Shaper A.G. (2008). The Value of Gamma-Glutamyltransferase in Cardiovascular Risk Prediction in Men without Diagnosed Cardiovascular Disease or Diabetes. Atherosclerosis.

[72] Bots M., Salonen J., Elwood P., Nikitin Y., Freire D., Concalves A., Inzitari D., Sivenius J., Trichopoulou A., Tuomilehto J. (2002). Gamma-Glutamyltransferase and Risk of Stroke: The Eurostroke Project. J. Epidemiol. Community Health.

[73] Emdin M., Passino C., Franzini M., Paolicchi A., Pompella A. (2007). γ-Glutamyltransferase and Pathogenesis of Cardiovascular Diseases. Future Cardiol..

[74] Franzini M., Corti A., Martinelli B., Del Corso A., Emdin M., Parenti G.F., Glauber M., Pompella A., Paolicchi A. (2009). γ-Glutamyltransferase Activity in Human Atherosclerotic Plaques—Biochemical Similarities with the Circulating Enzyme. Atherosclerosis.

[75] Koenig G., Seneff S. (2015). Gamma-Glutamyltransferase: A Predictive Biomarker of Cellular Antioxidant Inadequacy and Disease Risk. Dis. Markers.

[76] Parikh N.S., VanWagner L.B., Elkind M.S.V., Gutierrez J. (2019). Association between Nonalcoholic Fatty Liver Disease with Advanced Fibrosis and Stroke. J. Neurol. Sci..

[77] Calzadilla-Bertot L., Jeffrey G.P., Jacques B., McCaughan G., Crawford M., Angus P., Jones R., Gane E., Munn S., Macdonald G. (2019). Increasing Incidence of Nonalcoholic Steatohepatitis as an Indication for Liver Transplantation in Australia and New Zealand. Liver Transpl..

[78] Singal A.K., Hasanin M., Kaif M., Wiesner R., Kuo Y.F. (2016). Nonalcoholic Steatohepatitis Is the Most Rapidly Growing Indication for Simultaneous Liver Kidney Transplantation in the United States. Transplantation.

[79] Targher G., Bertolini L., Rodella S., Lippi G., Zoppini G., Chonchol M. (2010). Relationship between Kidney Function and Liver Histology in Subjects with Nonalcoholic Steatohepatitis. Clin. J. Am. Soc. Nephrol..

[80] Marcuccilli M., Chonchol M. (2016). NAFLD and Chronic Kidney Disease. Int. J. Mol. Sci..

[81] Seitz H.K., Mueller S., Hellerbrand C., Liangpunsakul S. (2015). Effect of chronic alcohol consumption on the development and progression of non-alcoholic fatty liver disease (NAFLD). Hepatobiliary Surg. Nutr..

[82] Persson E.C., Schwartz L.M., Park Y., Trabert B., Hollenbeck A.R., Graubard B.I., Freedman N.D., McGlynn K.A. (2013). Alcohol consumption, folate intake, hepatocellular carcinoma, and liver disease mortality. Cancer Epidemiol. Biomark. Prev..

[83] Morgan T.R., Mandayam S., Jamal M.M. (2004). Alcohol and hepatocellular carcinoma. Gastroenterology.

[84] Chalasani N., Younossi Z., Lavine J.E., Diehl A.M., Brunt E.M., Cusi K., Charlton M., Sanyal A.J. (2012). The Diagnosis and Management of Non-Alcoholic Fatty Liver Disease: Practice Guideline by the American Association for the Study of Liver Diseases, American College of Gastroenterology, and the American Gastroenterological Association. Am. J. Gastroenterol..

[85] Kirovski G., Schacherer D., Wobser H., Huber H., Niessen C., Beer K., Schoelmerich J., Hellerbrand C. (2010). Prevalence of Ultrasound-Diagnosed Non-Alcoholic Fatty Liver Disease in a Hospital Cohort and Its Association with Anthropometric, Biochemical and Sonographic Characteristics. Int. J. Clin. Exp. Med..

[86] Wannamethee S.G., Camargo C.A., Manson J.E., Willett W.C., Rimm E.B. (2003). Alcohol Drinking Patterns and Risk of Type 2 Diabetes Mellitus among Younger Women. Arch. Intern. Med..

[87] Vilar-Gomez E., Calzadilla-Bertot L., Wai-Sun Wong V., Castellanos M., Aller-de la Fuente R., Metwally M., Eslam M., Gonzalez-Fabian L., Alvarez-Quiñones Sanz M., Conde-Martin A.F. (2018). Fibrosis Severity as a Determinant of Cause-Specific Mortality in Patients with Advanced Nonalcoholic Fatty Liver Disease: A Multi-National Cohort Study. Gastroenterology.

[88] Cariello M., Piccinin E., Moschetta A. (2021). Transcriptional Regulation of Metabolic Pathways via Lipid-Sensing Nuclear Receptors PPARs, FXR, and LXR in NASH. Cell Mol. Gastroenterol. Hepatol..

[89] Petrick J.L., Campbell P.T., Koshiol J., Thistle J.E., Andreotti G., Beane-Freeman L.E., Buring J.E., Chan A.T., Chong D.Q., Doody M.M. (2018). Tobacco, alcohol use and risk of hepatocellular carcinoma and intrahepatic cholangiocarcinoma: The Liver Cancer Pooling Project. Br. J. Cancer.

[90] Benhammou J.N., Lin J., Hussain S.K., El-Kabany M. (2020). Emerging Risk Factors for Nonalcoholic Fatty Liver Disease Associated Hepatocellular Carcinoma. Hepatoma Res..

[91] Jadhav K., Cohen T.S. (2020). Can You Trust Your Gut? Implicating a Disrupted Intestinal Microbiome in the Progression of NAFLD/NASH. Front. Endocrinol..

[92] Jiang W., Wu N., Wang X., Chi Y., Zhang Y., Qiu X., Hu Y., Li J., Liu Y. (2015). Dysbiosis gut microbiota associated with inflammation and impaired mucosal immune function in intestine of humans with non-alcoholic fatty liver disease. Sci. Rep..

[93] Zhu L., Baker S.S., Gill C., Liu W., Alkhouri R., Baker R.D., Gill S.R. (2013). Characterization of gut microbiomes in nonalcoholic steatohepatitis (*NASH*) patients: A connection between endogenous alcohol and *NASH*. Hepatology.

[94] Satapathy S.K., Banerjee P., Pierre J.F., Higgins D., Dutta S., Heda R., Khan S.D., Mupparaju V.K., Mas V., Nair S. (2020). Characterization of Gut Microbiome in Liver Transplant Recipients with Nonalcoholic Steatohepatitis. Transplant. Direct.

[95] Nair S., Cope K., Risby T.H., Diehl A.M. (2001). Obesity and female gender increase breath ethanol concentration: Potential implications for the pathogenesis of nonalcoholic steatohepatitis. Am. J. Gastroenterol..

[96] Vallianou N., Christodoulatos G.S., Karampela I., Tsilingiris D., Magkos F., Stratigou T., Kounatidis D., Dalamaga M. (2021). Understanding the Role of the Gut Microbiome and Microbial Metabolites in Non-Alcoholic Fatty Liver Disease: Current Evidence and Perspectives. Biomolecules.

[97] Liu K., McCaughan G.W. (2018). Epidemiology and Etiologic Associations of Non-Alcoholic Fatty Liver Disease and Associated HCC. Adv. Exp. Med. Biol..

[98] Zoller H., Tilg H. (2016). Nonalcoholic fatty liver disease and hepatocellular carcinoma. Metabolism.

[99] Sandnes M., Ulvik R.J., Vorland M., Reikvam H. (2021). Hyperferritinemia-A Clinical Overview. J. Clin. Med..

[100] Jaruvongvanich V., Riangwiwat T., Sanguankeo A., Upala S. (2016). Outcome of phlebotomy for treating nonalcoholic fatty liver disease: A systematic review and meta-analysis. Saudi J. Gastroenterol..

[101] Beaton M.D., Chakrabarti S., Adams P.C. (2014). Inflammation is not the cause of an elevated serum ferritin in non-alcoholic fatty liver disease. Ann. Hepatol..

[102] Leite N.C., Salles G.F., Araujo A.L., Villela-Nogueira C.A., Cardoso C.R. (2009). Prevalence and associated factors of non-alcoholic fatty liver disease in patients with type-2 diabetes mellitus. Liver Int..

[103] Sookoian S., Pirola C.J. (2008). Non-alcoholic fatty liver disease is strongly associated with carotid atherosclerosis: A systematic review. J. Hepatol..

[104] Targher G., Day C.P., Bonora E. (2010). Risk of cardiovascular disease in patients with nonalcoholic fatty liver disease. N. Engl. J. Med..

[105] Targher G., Byrne C.D., Tilg H. (2020). NAFLD and increased risk of cardiovascular disease: Clinical associations, pathophysiological mechanisms and pharmacological implications. Gut.

[106] Mittal S., Sada Y.H., El-Serag H.B., Kanwal F., Duan Z., Temple S., May S.B., Kramer J.R., Richardson P.A., Davila J.A. (2015). Temporal Trends of Nonalcoholic Fatty Liver Disease–Related Hepatocellular Carcinoma in the Veteran Affairs Population. Clin. Gastroenterol. Hepatol..

[107] Chen Y.C., Li H., Wang J. (2020). Mechanisms of metformin inhibiting cancer invasion and migration. Am. J. Transl. Res..

[108] Mantovani A., Dalbeni A. (2021). Treatments for NAFLD: State of Art. Int. J. Mol. Sci..

[109] Ramai D., Singh J., Lester J., Khan S.R., Chandan S., Tartaglia N., Ambrosi A., Serviddio G., Facciorusso A. (2021). Systematic review with meta-analysis: Bariatric surgery reduces the incidence of hepatocellular carcinoma. Aliment. Pharmacol. Ther..

[110] Harrison S.A., Bashir M.R., Lee K.J., Shim-Lopez J., Lee J., Wagner B., Smith N.D., Chen H.C., Lawitz E.J. (2021). A structurally optimized FXR agonist, MET409, reduced liver fat content over 12 weeks in patients with non-alcoholic steatohepatitis. J. Hepatol..

[111] Jiang H., Chen H.C., Lafata K.J., Bashir M.R. (2021). Week 4 Liver Fat Reduction on MRI as an Early Predictor of Treatment Response in Participants with Nonalcoholic Steatohepatitis. Radiology.

